# Prevalence, characteristics and correlates of enteric pathogenic protozoa in drinking water sources in Molyko and Bomaka, Cameroon: a cross-sectional study

**DOI:** 10.1186/s12866-016-0890-5

**Published:** 2016-11-08

**Authors:** Fuh Anold Nsoh, Buh Amos Wung, Julius Atashili, Pokam Thumamo Benjamin, Eba Marvlyn, Keumami Katte Ivo, Assob Jules Clément Nguedia

**Affiliations:** 1 Faculty of Health Sciences, University of Buea, P.O. Box 63, Buea, Cameroon; 2 Department of Public Health and Hygiene, Faculty of Health Sciences, University of Buea, P.O. Box 63, Buea, Cameroon

**Keywords:** Enteric pathogenic protozoa, Drinking water, Prevalence, Buea, Cameroon

## Abstract

**Background:**

Access to potable water remains a major challenge particularly in resource-limited settings. Although the potential contaminants of water are varied, enteric pathogenic protozoa are known to cause waterborne diseases greatly. This study aimed at investigating the prevalence, characteristics and correlates of enteric pathogenic protozoa in drinking water sources in Buea, Cameroon.

**Methods:**

A cross-sectional study was conducted using 155 water samples collected from various drinking sources (boreholes, springs, taps and wells). Each sample was subjected to physicochemical examinations (pH, turbidity, odour and sliminess) and parasitological analysis (wet mount, modified Ziehl-Neelsen stain) to determine the presence of enteric pathogenic protozoa. A data collection tool was used to note characteristics of collected samples and the data was analysed using EPI-INFO Version 3.5.3.

**Results:**

The overall prevalence of enteric pathogenic protozoa in water sources was 62.6 %. Eight species of enteric protozoa were observed with *Cryptosporidium parvum* being the most predominant (45.8 %). Spring water was the most contaminated source with enteric protozoa (85.7 %) while pipe borne water had all eight species of protozoa identified. A pH of 6 was the only significant factor associated with the prevalence of these pathogens in water sources.

**Conclusion:**

The prevalence of enteric protozoa in water sources in Molyko and Bomaka is high, spring water is the most contaminated water source and *Cryptosporidium parvum* is the most common protozoa contaminating water. A water pH of 6 is associated to the prevalence of protozoa. Community members need to be educated to treat water before drinking to avoid infection by enteric protozoa in water and further studies with larger samples of water need to be conducted to find other correlates of the presence of protozoa in water.

## Background

Water covers almost 70 % of the earth’s surface but most water sources are saline with the drinkable type (fresh water) constituting only 2.5 % of the earth’s water [1]. Access to drinking water is not only important for health but also for sustainable development, food production and poverty reduction [2]. Despite its importance, access to safe drinking water remains a challenge worldwide. In 2010, reports showed that safe drinking water remained inaccessible to over 1.1 billion people in the world with about 400 deaths of children below the age of five arising every hour due to biological contamination of water [3].

Contaminants of water can either be microorganisms (bacteria, protozoa, helminths and viruses) or chemicals [4]. Microorganisms are often found in water as a result of sewage discharges (containing faecal matter), leaking septic tanks and runoffs from animal feedlots [5]. Chemical contaminants on the other hand, are usually considered a lower priority contaminant since their adverse health effects are generally associated with long term exposure whereas the effects of microbial contaminants are usually immediate [6].

Enteric protozoa are a major waterborne pathogen in developing countries causing diarrhoeal illnesses in humans, with some causing a severe debilitating illness that can shorten the lifespan of an immune compromised individual [7]. Bacterial contamination, however, contributes to the largest share of the diarrhoea disease burden associated with unsafe drinking water with children less than 5 years of age being very vulnerable [8].

The types of enteric protozoa that can be found in water are diverse. In developing settings such as sub-Saharan Africa and Asia, the common enteric pathogenic protozoa that can be found in water include *Entamoeba* species (spp), *Cryptosporidium* spp*, Giardia intestinalis, Microsporidia* species and *Cyclospora cayetanensis* [9, 10]. Other species such as *Blastocystis* spp and *Dientamoeba fragilis* are usually isolated in developed countries [9].

In sub-Saharan Africa, the transmission of these enteric parasites in water is favoured by socio-economic factors such as poor hygiene, lack of safe water and sanitation facilities. Low socio-economic status is known to play a pivotal role in susceptibility to infection [11]. Secondly, most of the enteric parasites have the ability to complete their life-cycles within a single host that excretes large numbers of infective transmissible stages (such as *Giardia* cysts and *Cryptosporidium* oocysts) in faeces. Also, zoonotic transmission of enteric protozoa enhances both the reservoir of infection and environmental contamination, thereby increasing the likelihood of waterborne transmission [12].

Some studies in Africa have shown the presence of protozoa in water sources. In Nigeria for example, a study [13] on parasites in domestic water sources reported a prevalence of enteric pathogenic protozoa of 42.4 % while in Zimbabwe, a prevalence of enteric pathogenic protozoa of 36.6 % was reported [14]. In Ghana, a prevalence of up to 77.8 % of enteric protozoa in water samples was reported [10].

However, in Cameroon, no study has been carried out on the prevalence of enteric pathogenic protozoa in drinking water sources. Instead, studies on these pathogens have used stool samples of patients attending hospitals [15] and food vendors [16]. The objective of this study therefore was to provide data on the prevalence, different species of protozoa and the factors associated with the prevalence of these species in drinking water sources in the Molyko and Bomaka communities in Buea.

## Methods

### Study design and setting

A cross-sectional, descriptive and analytic study was conducted on water samples collected in Molyko (an urban community) and Bomaka (a semi-urban community) both found in Buea, Cameroon.

Briefly, Buea is a town and capital of the South-West Region. It covers a surface area of about 870 km^2^, with a population of 200,000 [17]. The town is located at the base of Mount Cameroon and has 85 villages. Buea is renowned for its high-quality groundwater percolating into the volcanic sub-strata of the mountain and surfacing as natural springs in various locations towards the middle and lower regions of the town. These ‘traditional’ water sources supplement public supply, and are heavily used by the town’s inhabitants for activities ranging from drinking, to construction, and the washing of vehicles. Inhabitants of Molyko predominantly use pipe borne water either from the national water supplier (CAMWATER) or community water supplies. Very few boreholes are found in this area. Bomaka on the other hand does not have national water supplies and its dwellers use boreholes, springs, wells or community pipe-borne water supplies.

### Study sample and sampling

The study samples were drinking water samples collected from taps, wells, boreholes and springs from June 2014 to August 2014. The sample size was determined using a formula for estimating population proportions for a cross-sectional study [18]. We assumed the prevalence of enteric pathogenic protozoa in drinking water sources to be 42.4 % as reported in a study in Nigeria [13], a 95 % confidence level and an error margin of 7 %. This gave a sample size of 192. However, 155 samples were collected because most quarters in Bomaka shared a common drinking source.

Convenience sampling was done by dividing Molyko and Bomaka respectively into 14 and 7 quarters. To estimate the number of samples to be collected from each quarter, the 155 sample size was divided by the 21 quarters in the study. This gave seven water samples per quarter.

### Data and sample collection

Data collection was done by one of the investigators using forms to record information on some characteristics which could influence prevalence of protozoa in water such as the water supplier (national, community, individual), source of water (river, stream, spring, well, borehole, tap), location of water source (open air or in a closed environment), exposure of pipes to the surface, presence or absence of a lid on the water source and distance of the water source from animal contact, toilets, pit latrines, septic tanks and farms. We also collected information on how often these water sources were cleaned and observed the physical environment that surrounded these water sources.

Clean bottles were used to collect water samples at consumption points for physicochemical and parasitological analysis. All samples collected were labelled with date of collection, identification number and site of collection. The information collected was then registered in a sampling and parasitological analysis form and transported to the Faculty of Health Sciences’ Teaching Laboratory at the University of Buea for analysis.

### Physicochemical and parasitological analysis/measurements

Physicochemical analysis of the water samples involved the measurement of the pH, turbidity, odour and sliminess. Measurement of pH was done using Whatman™ 2600-100A pH test paper. Turbidity, odour and sliminess were evaluated using sense organ perceptions by the investigator during collection of the water sample at its source following recommended methods [14].

With regards to parasitological analysis, each collected sample was shaken and the cap of the water bottle carefully removed avoiding touching the opening with bare hands. The bottle contents were dispensed into falcon test tubes and centrifuged at 2000 rotations per minute (rpm) for 5 minutes. Then, the contents of each of these test tubes were pooled together after the supernatant fluid had been discarded. The combined sediment were re-centrifuged under the same conditions as above and the supernatant fluid was again discarded for the sediments to be examined microscopically as recommended by Kwakye-Nuako and colleagues [10]. Direct wet/iodine preparation for identification of cysts was done using the method described by the WHO [19] while Modified Ziehl-Neelsen technique was done to detect the presence of other cysts and coccidian oocysts as documented by Kwakye-Nuako and colleagues [10].

### Data management and statistical analysis

All data from the data collection form were keyed into an Epi Info database (WHO/CDC Atlanta, USA) and analysed using Epi Info version 3.5.3, Statistical package for Social Sciences (SPSS) Statistics 20 and Microsoft excel 2007. Water samples were described using frequencies and percentages of the water sources from which they were collected and the species and number of water samples with enteric pathogenic protozoa.

The prevalence of enteric pathogenic protozoa in the drinking water sources was obtained by computing the ratio of the number of water samples containing protozoa and the total number of water samples analysed (expressed as a percentage).

To characterise the pathogens in terms of species, the ratio of the number of water samples with a pathogen of interest and the total number of samples which contain enteric pathogenic protozoa (expressed as a percentage) was calculated.

To determine the factors associated with the presence of enteric pathogenic protozoa in water sources, bivariate and multivariate analysis was done. Bivariate analysis was done by considering the prevalence of protozoa as a binary outcome variable and the characteristics of water sources as predictors. Unadjusted odd ratios, 95 % confidence intervals and p-values were computed. All variables with *p*-values ≤ 0.2 were considered as having a potential association to prevalence of protozoa and were considered for further analysis in a multivariate logistic regression model to check for confounders. The multivariate analysis was done by considering prevalence of protozoa as a binary outcome variable and variables with p-values ≤ 0.2 in the bivariate analysis as predictors. Adjusted odd ratios, 95 % confidence intervals and p-values were also computed. Any variable that had a p-value <0.05 was considered as having a statistically significant association with the prevalence of enteric pathogenic protozoa in water sources.

## Results

### Water sources and sample characteristics

Of the 155 water samples analysed 51 % was collected from the national water supplier source. Most (68.1 %) of the water samples collected in Molyko were from the national water supplier while 53.8 % of the water samples in Bomaka were obtained from the community water supplier source. Overall, 77.4 % of water samples were collected from taps (Table 1).

**Table 1:** Distribution of 155 water samples based on place of collection and water suppliers/sources

	Place of collection		Total (%)
	Molyko	Bomaka	
Water suppliers			
*National (%)*	79 (68.1)	0 (0.0)	79 (51.0)
*Community (%)*	28 (24.1)	21 (53.8)	49 (31.6)
*Individual (%)*	9 (7.8)	18 (46.2)	27 (17.4)
Water sources			
*Borehole (%)*	14 (12.1)	6(15.4)	20 (12.9)
*Spring (%)*	2 (1.7)	5 (12.8)	7 (4.5)
*Tap (%)*	100 (86.2)	20 (51.3)	120 (77.4)
*Well (%)*	0 (0.0)	8 (20.5)	8 (5.2)

*%* percentage

Most water sources such as boreholes and wells constructed outside homes (67.1 %) were uncovered and 91 % of them were constructed at least one metre above the ground. Over 30 % of pipe borne water sources had pipes exposed to the surface. Most of the sources (92.3 % and 82.6 %) were located more than 10 metres away from toilets or pit latrines and septic tanks respectively. Seventy (45.2 %) of the water sources had existed for at most 5 years and mud surrounded 39.4 % of the water sources even though 51.6 % of the water sources were reported to be cleaned weekly. The predominant pH of most water sources (80.6 %) ranged between 6 and 7 (Table 2).

**Table 2:** Characteristics of water sources of the 155 collected samples

Characteristics of water source	Frequency (N)	Percentage (%)
Location of the water source		
Open (outdoor)	104	67.1
Closed (indoor)	51	32.9
Lid on water source		
Present	29	18.7
NA*	126	81.3
Height above the ground		
≤ 1 metre	10	6.5
> 1 metre	141	91.0
NA*	4	2.6
Pipes exposed		
Not exposed	95	61.3
Exposed	47	30.3
NA	13	8.4
Distance from toilet or pit latrine		
0–1 m	3	1.9
2–10 m	9	5.8
> 10 m	143	92.3
Distance from septic tanks		
0–1 m	1	0.6
2–10 m	26	16.8
> 10 m	128	82.6
Distance from farms		
0–1 m	17	11.0
2–10 m	22	14.2
> 10 m	116	74.8
Animals around source		
0–1 m	2	1.3
2–10 m	1	0.6
> 10 m	155	98.1
Existence of source		
0–5 years	70	45.2
6–10 years	37	23.9
> 10 years	48	31.0
Mud around source		
Absent	94	60.6
Present	61	39.4
Covered by cement		
Absent	46	29.7
Present	109	70.3
Stagnant water		
Absent	98	63.2
Present	57	36.8
Enter water with feet		
No	2	1.3
Yes	5	3.2
NA*	148	95.5
Cleaning frequency		
Never or rarely	19	12.3
Weekly	80	51.6
Monthly	51	32.9
Yearly	5	3.2
pH		
6	30	19.4
7	125	80.6
Turbidity		
Clear	141	91.0
Cloudy	2	1.3
Brownish	2	1.3
Contain particles	10	6.5
Odour		
No	145	93.5
Yes	10	6.5
Sliminess		
Not slimy	145	93.5
Slimy	10	6.5

*N* frequency, NA* not applicable to the given characteristic, *%* percentage

### Prevalence and characterisation of enteric pathogenic protozoa

**Fig. 1:**
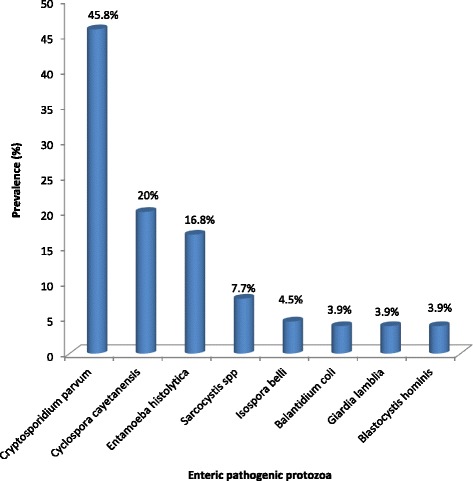
Prevalence of individual enteric pathogenic protozoa observed in water samples. The data in this figure presents the prevalence of the protozoa that were observed in the water samples (*Cryptosporidium parvum (45.8 %), Cyclospora cayetanensis (20 %), Entamoeba hystolytica (16.8 %), Sarcocystis spp (7.7 %), Isospora belli (4.5 %), Balantidium coli (3.9 %), Giardia lamblia (3.9 %), Blastocystis hominis (3.9 %))*

Of the 155 water samples examined, 97 (62.6 %) harboured enteric pathogenic protozoa. Eight species of enteric pathogenic protozoa were observed with *Cryptosporidium parvum* oocytes (45.8 %) being the most common (Fig. 1). Water from springs had the highest degree of contamination (85.7 %) with enteric pathogenic protozoa (Table 3). Most of these spring water sources (71.4 %) mainly harboured *Cryptosporidium parvum.* Only tap water harboured all the eight species of enteric pathogenic protozoa observed (Table 4).

**Table 3:** Rate of contamination of water sources by enteric pathogenic protozoa

Water source	Number of samples collected	Number of positive samples	(%)
Borehole	20	12	60.0
Spring water	7	6	85.7
Tap water	120	77	64.2
Well	8	2	25.0
Total	155	97	62.6

*%* percentage

**Table 4:** Distribution of enteric pathogenic protozoa in the 4 different water sources

Water source (*n* = 155)	CP (%)	CC (%)	EH (%)	Sarco (%)	IB (%)	BC (%)	GL (%)	BH (%)	Total *(%)
Boreholes (*n* = 20)	9 (45.0)	8 (40.0)	0 (0.0)	3 (15.0)	1 (5.0)	1 (5.0)	1 (5.0)	2 (10)	25 (15.3)
Springs (*n* = 7)	5 (71.4)	0 (0.0)	3 (42.9)	0 (0.0)	1 (14.3)	0 (0.0)	1 (14.3)	0 (0.0)	10 (6.1)
Taps (*n* = 120)	57 (47.5)	21 (17.5)	21 (17.5)	9 (7.5)	5 (4.2)	5 (4.2)	4 (3.3)	4 (3.3)	126 (77.3)
Wells (*n* = 8)	0 (0.0)	1 (12.5)	1 (12.5)	0 (0.0)	0 (0.0)	0 (0.0)	0 (0.0)	0 (0.0)	2 (1.2)
Total**	71 (45.8)	30 (19.4)	25 (16.1)	12(7.7)	7 (4.5)	6 (3.9)	6 (3.9)	6 (3.9)	163

CP *Cryptosporidum parvum*, CC *Cyclospora cayetanensis,* EH *Entamoeba histolytica,* Sarco *Sarcocystis spp,* IB *Isospora belli,* BC *Balantidium coli*, GL *Giardia lamblia*, BH *Blastocystis hominis,* Total* = total number of pathogens identified, Total** = total number of individual species of protozoa in 155 water samples

### Correlates of enteric pathogenic protozoa in water sources

Tables 5 and 6 presents the correlates of enteric pathogenic protozoa in water sources. In the bivariate analysis, the factors that appeared to have an association with the prevalence of enteric pathogenic protozoa in water sources include having opened water sources when compared to closed ones, having water sources out of homes with no lids when compared to sources that do not need lids, having water sources with exposed pipes when compared to sources with unexposed pipes, having water sources ≤10 m away from septic tanks when compared to sources >10 m away from septic tanks, having water sources that have existed for more than 5 years when compared to sources with five or less years of existence, having mud around water sources when compared to sources with no mud surrounding them, having water sources with non-cemented surroundings, having water sources that are rarely/never cleaned when compared to sources that are at least cleaned weekly/monthly, having water sources with a pH of 6 when compared to sources with a pH of 7, having water sources with a cloudy turbidity when compared to sources with a clear turbidity, having water sources with an odour when compared to sources with no odour and having slimy water sources when compared to sources that are not slimy (Table 5).

**Table 5:** Correlates of enteric pathogenic protozoa in water sources-bivariate analysis

Predictor	N	%	OR*	95 % CI	*p*-value
Location of water source					
Closed	28	54.9	Ref		
Open	69	66.3	1.62	0.82–3.21	0.16
Lid on water source					
NA	87	65.1	Ref		
Yes	15	51.7	0.57	0.25–1.30	0.18
Height above ground level					
≤ 1 m	6	60.0	Ref		
> 1 m	88	62.4	1.11	0.30–4.10	0.88
NA	3	75.0	2.00	0.15–26.72	0.60
Pipes exposed					
Not exposed	56	58.9	Ref		
Exposed	35	74.5	2.03	0.94–4.39	0.07
NA	6	46.2	0.60	0.19–1.91	0.40
Distance from toilet					
≤ 1 m	2	66.7	Ref		
2–10 m	7	77.8	1.75	0.10–30.82	0.70
> 10 m	88	61.5	0.80	0.07–9.03	0.86
Distance from septic tank					
> 10 m	77	60.2	Ref		
≤ 10 m	20	74.1	1.89	0.75–4.80	0.17
Distance from farm					
≤ 1 m	11	64.7	Ref		
2–10 m	14	63.6	0.95	0.25–3.57	0.94
> 10 m	72	62.1	0.89	0.31–2.58	0.83
Animals around source					
> 10 m	94	61.8	Ref		
≤ 10 m	3	100.0	0.00	0.00- > 1.0E12	0.24
Existence of water source					
≤ 5 years	51	72.9	Ref		
6–10years	20	54.1	0.44	0.19–1.01	0.05
> 10 years	26	54.2	0.44	0.20–0.96	0.04
Mud					
Present	42	68.9	Ref		
Absent	55	58.5	0.64	0.32–1.26	0.19
Covered by cement					
Present	64	58.7	Ref		
Absent	33	71.7	1.78	0.85–3.77	0.13
Stagnant water					
Present	39	68.4	Ref		
Absent	58	59.2	0.67	0.33–1.33	0.25
Enter water with feet					
Yes	4	80.0	Ref		
No/NA	93	62.0	0.41	0.04–3.74	0.38
Cleaning frequency					
Never/rarely	17	89.5	Ref		
Weekly	46	57.5	0.16	0.03–0.73	0.02
Monthly	31	60.8	0.18	0.04–0.87	0.03
Yearly	3	60.0	0.18	0.02–1.78	0.14
pH					
7	72	57.6	Ref		
6	25	83.3	3.68	1.32–10.24	0.01
Turbidity					
Cloudy/contain particles	11	78.6	Ref		
Clear	86	61.0	0.43	0.11–1.60	0.19
Odour					
Yes	9	90.0	Ref		
No	88	60.7	0.17	0.02–1.39	0.06
Sliminess					
Yes	4	40.0	Ref		
No	93	64.1	2.68	0.72–9.94	0.12

*OR unadjusted odds ratio, *OR* odds ratio, *N* frequency of positive sample, *%* percentage of positive samples, *NA* not applicable to the factor being studied, *Ref* reference variable category, *CI* confidence interval, *p*-values ≤0.2 suggests a possible association to the presence of enteric pathogenic protozoa

**Table 6:** Correlates of enteric pathogenic protozoa in water sources-multivariate analysis

Predictor	aOR*	95 % CI	*p*-value
Location of water source			
Open	Ref		
Closed	1.85	0.61–5.55	0.28
Lid on water source			
Yes	Ref		
NA	1.63	0.34–7.73	0.54
Pipes exposure			
Not exposed	Ref		
Exposed	1.93	0.74–5.04	0.17
NA	0.46	0.04–5.72	0.54
Distance from septic tank			
≤ 10 m	Ref		
> 10 m	0.51	0.17–1.53	0.23
Distance from animals			
≤ 10 m	Ref		
> 10 m	0.00	0.00- > 1.0E12	0.96
Existence of water source			
≤ 5 years	Ref		
6–10years	0.55	0.15–1.95	0.35
> 10 years	0.47	0.14–1.64	0.24
Mud			
Absent	Ref		
Present	1.44	0.54–3.87	0.45
Covered by cement			
Absent	Ref		
Present	0.56	0.20–1.58	0.27
Cleaning frequency			
Never/rarely	Ref		
Weekly	0.15	0.02–1.19	0.07
Monthly	0.20	0.03–1.51	0.12
Yearly	0.19	0.01–3.73	0.28
pH			
6	Ref		
7	0.27	0.09–0.83	0.02**
Turbidity			
Clear	Ref		
Cloudy/contain particles	1.98	0.40–9.75	0.40
Odour			
No	Ref		
Yes	4.06	0.41–40.54	0.23
Sliminess			
No	Ref		
Yes	1.59	0.10–24.29	0.74

*aOR adjusted odds ratio, *OR* odds ratio, *ref* reference variable category

**bold = statistically significant result, *NA* not applicable to the factor being studied, *Ref* reference variable category, *CI* confidence interval, *p*-values < 0.05 are statistically significant

After adjusting for potential confounding by each of the water sources factors that appeared to have an association with prevalence of enteric pathogenic protozoa in the bivariate analysis, only having water sources with a pH of 6 remained a statistically significant predictor of prevalence of enteric pathogenic protozoa in water sources. In fact, the odds of having enteric pathogenic protozoa in water sources with pH = 7 was 0.27 times (95 % CI: 0.09-0.83) that in water sources with pH = 6 (Table 6).

## Discussion

Nowadays, the consumption of water in most developing countries is based on its aesthetic quality with little or no attention paid on its microbiological or chemical quality [20]. It has however been noted that most waterborne infections and deaths in developing countries arise from parasitic diseases. It is thus important to investigate the safety of water used for human consumption [9].

In this study, to appraise the presence of enteric pathogenic protozoa in drinking water sources, we assessed the prevalence of enteric protozoa, characterised the species of protozoa found in drinking water sources and also assessed the association between prevalence of protozoa and water sources’ characteristics. We document that the prevalence of enteric pathogenic protozoa in water sources is high. Approximately, 63 % of drinking water sources are infested with enteric pathogenic protozoa-too high a percentage per se, to be less concerned about. We also document that eight species of enteric pathogenic protozoa were identified in drinking water sources. These include *Cryptosopridium parvum, Cyclospora cayetanensis, Entamoeba histolytica, Giardia lamblia*, *Sarcocystis spp, Blastocystis hominis, Isospora belli* and *Balantidium coli. Cryptosporidium parvum* was the most predominant enteric parasite identified, spring water was observed to be the most contaminated water source and tap water had all the types of enteric pathogenic protozoa observed. In the study samples, available characteristics of water sources both individually and as a group did not accurately distinguish water sources having enteric protozoa and those that did not. However, water sources with a pH of 6 had statistically significant associations with prevalence of enteric protozoa.

While the prevalence of enteric pathogenic protozoan organisms in drinking water sources in Molyko and Bomaka appears high (62.6 %), it is within the range of prevalence of enteric pathogenic protozoa reported in similar studies done elsewhere. We found no studies on the prevalence and characterisation of enteric pathogenic protozoa in drinking water sources in Cameroon before this study, but the prevalence of enteric protozoa in sub-Saharan Africa has ranged from 36.6 to 77.8 % in Nigeria [13], Zimbabwe [14] and Ghana [10]. The highest prevalence of enteric pathogenic protozoa (77.8 %) we found so far was reported in a study in Ghana [10].

The prevalence of enteric pathogenic protozoa in this study could be influenced by differences in the characteristics of water sources from which samples were collected. The collected water samples for this study may not have been representative of all drinking water sources in the quarters. However, we do not expect the difference in prevalence to be substantial as samples were collected in all quarters of Molyko and Bomaka (the study area). Potential errors due to bias in sample collection or poor sample collection could mean that our prevalence of enteric pathogenic protozoa in water sources is over estimated and that our not finding an association between prevalence and many water resources’ characteristics is misleading. Nevertheless, the quality of our sample was assured by using one of the investigators to collect water samples for this study in well cleaned containers. Also, the nature of this study’s technique did not allow for a quantification of the number of parasites in a unite volume of water-making it difficult to know the possibility of getting infected if one drinks a glass of water for example. However, the results do not only allow a comparison between different water sources but also allows people to choose to avoid one source over another if they cannot sterilise their water.

The identification of protozoa in all water supply sources-borehole (60.0 %), spring water (85.7 %), tap water (64.2 %) and well water (25.0 %) confirms that no water supply source is safe for drinking no matter how clean or clear its water appears. This reiterates the need to teach community dwellers how to treat water before drinking and the need for water suppliers to revise their water treatment strategies before distributing water for community consumption.

We found no studies done in Cameroon assessing prevalence of enteric pathogenic protozoa in drinking water sources. Most studies conducted were done on stool specimens of patients in hospital [15] and food vendors [16]. While our sample was adequate for estimating the prevalence of enteric pathogenic protozoa in drinking water sources, only a limited number of covariates appeared to have an association with prevalence of enteric protozoa and could be considered as potential predictors. This study showed a significant association between the pH of the water sources and the presence of enteric pathogenic protozoa. According to WHO guidelines, the normal pH range for potable water is 6.5–8.5. Enteric pathogenic protozoa were more prevalent in water sources that had pH = 6 than those with pH = 7 implying that they are found in a slightly acidic milieu than in neutral milieu. This is dissimilar to the results of a study carried out by Tremaine et al. [21], where the prevalence of protozoa was found to be associated to strong acidic or lower pH media but similar to the study conducted by Johnson et al. [22], which reported that the prevalence of protozoa is related to weak acidic media.

## Conclusion

The prevalence of enteric protozoa in water sources in Molyko and Bomaka is high, spring water is the most contaminated water source and *Cryptosporidium parvum* is the most common protozoa contaminating water. A water pH of 6 is associated to the prevalence of protozoa. Community members need to be educated to treat water before drinking to avoid infection by enteric protozoa in water and further studies with larger samples of water need to be conducted to find other correlates of the presence of protozoa in water.

## Abbreviations

aOR: Adjusted odds ratio
CAMWATER: Cameroon Water Utilities Corporation
CI: Confidence interval
ml: Millilitre
N: Frequency
OR: Odds ratio
Ref: Reference variable
rpm: Rotations per minute
SD: Standard deviation
spp: Species
UNESCO: United Nations Educational, Scientific and Cultural Organisation
USA: United States of America
WHO: World Health Organisation
